# Modeling Dent Disease Type 1 in Flies

**DOI:** 10.34067/KID.0000000000000441

**Published:** 2024-05-30

**Authors:** Franca Anglani, Giovanna Priante

**Affiliations:** Department of Medicine, Nephrology Unit, Kidney Histomorphology and Molecular Biology Laboratory, University of Padua, Padova, Italy

**Keywords:** cell biology and structure, Dent disease, endocytosis, hypercalciuria, kidney stones, molecular genetics, podocyte, proteinuria, renal proximal tubule cell, tubular epithelium

Dent disease is an X-linked recessive nephropathy characterized by proximal tubular dysfunction. Around 50%–60% of patients carry pathogenic variants in the *CLCN5* gene (Dent disease type 1 [DD1] Online Mendelian Inheritance in Man 300009), and 10%–15% of patients carry pathogenic variants in the *OCRL* gene (Dent disease type 2 Online Mendelian Inheritance in Man 300555). The remaining patients lack mutations in both of these genes.

**Figure 1:**
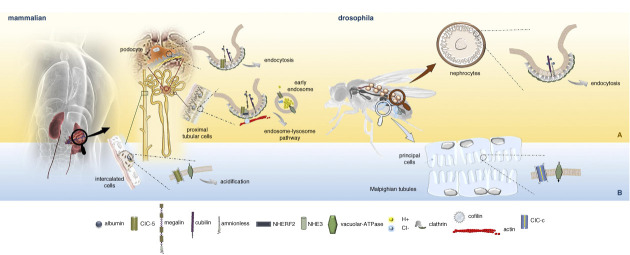
**Similarity between mammalian and Drosophila renal excretory structure and function: the macromolecular system for protein uptake**. (A) Cubilin and AMN, the two major receptors for albumin reabsorption present in the PT and podocytes along with megalin and ClC-5, were also identified in Drosophila nephrocytes, which in this way combine protein filtration and reabsorption in a single structure. Clc-c was not detected in nephrocytes. (B) The study by Reynolds *et al.*^8^ identifies ClC-c both in the principal cells of the MT main segment, which bear functional similarity to the intercalated cells of the mammalian collecting duct, where ClC-5 was also identified, and in those of the MT near the ureter, where a reabsorptive function was hypothesized. AMN, amnionless; ClC-c, chloride (Cl(-)) channel-c; ClC-5, chloride (Cl(-)) channel-5; MT, Malpighian tubule; PT, proximal tubule.

Both chloride (Cl(-)) channel-5 (ClC-5) and OCRL1 proteins have important functions in the endolysosomal pathway of proximal tubular cells (PTCs). ClC-5 localizes to the early endosome and is thought to use the proton gradient generated by the proton pump vacuolar H^+^ ATPase (V-ATPase) to transport chloride into the vesicular lumen. Chloride ions, which are transported in exchange with protons by ClC-5, may help to neutralize the positive charge inside the lumen generated by the proton pump. In PTCs, the contribution of ClC-5 to the acidic pH within the endosomes is important for ligand:receptor dissociation, subsequent recycling of the receptor to the apical membrane, and degradation of the ligand within endosomes.^1^ Experimental evidence indicates that the endosomal Cl^−^ concentration might play a role in endocytosis, independently of endosomal acidification, thus suggesting another possible mechanism by which ClC-5 dysfunction may impair endocytosis. ClC-5 may have additional functions because approximately 8% is located at the PTC plasma membrane. Here, it plays a key role in the formation/function of the endocytic complex that includes megalin and cubilin/amnionless (AMN) complex, the two main receptors for albumin and low–molecular weight (LMW) proteins, sodium-hydrogen antiporter 3, Na^+^-H^+^ exchanger regulatory factor 2, and V-ATPase (Figure 1A). Cells lacking ClC-5 show downregulation of two main receptor megalin and cubilin and consequently decreased endocytic uptake of albumin and LMW proteins.^1^

Although it is widely accepted that the triad of symptoms LMW proteinuria, hypercalciuria, and nephrocalcinosis/nephrolithiasis is pathognomonic of DD1, there is marked heterogeneity in the clinical presentation of the disease. With the exception of LMW proteinuria, which is the clinical sign shared by almost all patients, proximal tubular dysfunction manifests differently, and nephrocalcinosis and/or kidney stones as well as progressive renal failure affect around 50% and 30% of patients, respectively.^2^

Mouse models of DD1 were generated to explore the pathogenesis of the disease. In particular, the two independent *Clcn5* knockout mice strains, the so-called Jentsch^3,4^ and Guggino models,^5–7^ provided critical insights into the mechanisms of proximal tubule (PT) dysfunction. These two strains, both having the same genetic background, recapitulated the major features of the disease. Studies in the two models demonstrated that *Clcn5* inactivation is associated with severe impairment of both fluid phase and receptor-mediated endocytosis and a trafficking defect with the loss of megalin and cubilin at the PT brush border. However, targeted disruption of *Clcn5* in the Jentsch model did not lead to hypercalciuria, one of the hallmark of Dent disease that is present in approximately 80% of patients with DD1, while a similar disruption in the Guggino model led to hypercalciuria and nephrocalcinosis.^5^ How ClC-5 loss-of-function in PT could cause elevated urinary calcium is still not completely understood. Thus, the relationship between this 2Cl^−^/H^+^ transporter and Ca^2+^ metabolism remains to be elucidated.

The current paper by Reynolds *et al.*^8^ in this issue of *Kidney360* provides evidence for a novel *in vivo* model of DD1 based on a fly, the Drosophila melanogaster. The authors identified chloride (Cl(-)) channel-c (ClC-c) as the Drosophila homolog of human ClC-5 and established the world's first DD1 model by Malpighian tubule (MT)-specific knock-down of ClC-c. In this model, the knock-down of ClC-c caused an increase in urinary proteins and Ca^2+^ secretion as well as an increase in the appearance of spontaneous tubule crystals, thus mimicking the triad of symptoms typically observed in DD1 patients. Certainly, in humans, Dent disease is characterized by intricate molecular pathways and interactions that may not be fully replicated in flies, which could oversimplify the mechanisms involved in the disease. It is also possible that fly models do not fully capture the effects of specific variants associated with Dent disease, which could limit their utility for studying genotype–phenotype relationships.

That drosophila could be a reliable genetic model for studying kidney development and function is not unknown. A very recent review^9^ highlights the usefulness of this fly in kidney research for its easy genetics manipulations, including facility and low cost of maintenance, and less onerous regulation, as compared with mammalian systems. Unsuspectingly, the mammalian kidney with its complex and still elusive structure and function has in the fly a good kidney model. The fly model offers a clear analogy with the mammalian system in terms of the properties to be studied; the tissue is easily accessible, allowing the complexity of the organ to be addressed.

Circulating hemolymph is filtered by nephrocytes. It is known that the Drosophila nephrocyte and the mammalian glomerular podocyte are similar in filtration, but it remained uncertain whether there is an organ or cell type in flies that can reabsorb proteins. Zhang *et al.*^10^ demonstrated that the Drosophila nephrocyte has structural, functional, and molecular similarities to the renal PT (Figure 1A). The authors identified two Drosophila genes encoding mammalian cubilin and AMN orthologs whose expression is specific to nephrocytes, where they function as co-receptors for protein uptake. Furthermore, they also found that cubilin/AMN-mediated protein reabsorption is required for the maintenance of nephrocyte ultrastructure and fly survival under conditions of toxic stress. These results indicate that the nephrocyte, which brings together filtration and protein reabsorption, can serve as a simplified model for both podocytes and renal PTs. Further corroborating this similarity, megalin, cubilin, and ClC-5 were also discovered in human podocytes that share a close proximity with PTCs at the tubular pole of the glomerulus.^1^

MT is the fly renal tubule that secretes solutes from the hemolymph and produce urine similarly to renal epithelia. The main tubule segment comprises large principal cells and smaller stellate cells and generates primary urine through active transport rather than ultrafiltration. The lower tubule—the region between the main segment and the ureter—is assumed to be reabsorptive in analogy to the mammalian PT, but research on this segment is limited. Physiologically, MT secretes fluid faster than any other tissue and is well suited for investigating transport or secretory phenotypes, such as nephrolithiasis.^9^

In the mammalian kidney, ClC-5 is also expressed in the alpha-intercalated cells of collecting ducts. These cells secrete H^+^ into the urine through apical membrane V-ATPase while beta-intercalated cells export H^+^ into the vessel lumen through basolateral membrane V-ATPase, regulating final urine acidification.^1^ In the Drosophila, similarly to the collecting duct, the two main MT secretory cell types, the principal cell and the stellate cell, perform distinct transport roles: Principal cells drive electrogenic cation transport through V-ATPase, and stellate cells provide a shunt pathway for Cl^−^ and osmosis-driven water movement. The partitioning of the V-ATPase into specialized cell types strongly recall the specialized role of intercalated cells in the human kidney collecting duct.^9^

Reynolds *et al.*^8^ added a new piece of knowledge on the similarity between the function of MT and those of renal tubules. By voltage clamp experiments, they demonstrate that ClC-c is voltage-gated with Cl^−^-dependent and pH-sensitive currents as is ClC-5 in PT. *In vivo* expression of ClC-c-eGreen Fluorescent Protein in MT reveals that ClC-c localizes to the apical and subapical membrane of microvilli, as does ClC-5 in PT (Figure 1B). Furthermore, genetic manipulation of ClC-c (site direct mutagenesis to introduce homologous *CLCN5* mutations and RNAi knock-down) induced an impairment of ClC-c ion transport activity and calcium and protein urinary loss, as ClC-5 loss-of-function does in DD1 tubulopathy.
